# Revisiting the structure of the Yale Global Tic Severity Scale (YGTSS) in a sample of Chinese children with tic disorders

**DOI:** 10.1186/s12888-021-03399-5

**Published:** 2021-08-09

**Authors:** Fang Wen, Yi Gu, Junjuan Yan, Jingran Liu, Fang Wang, Liping Yu, Ying Li, Yonghua Cui

**Affiliations:** Department of Psychiatry, Beijing Children’s Hospital, Capital Medical University, National Center for Children’s Health, 56 Nanlishi Road, Beijing, 100101 China

**Keywords:** YGTSS, Reliability, Validity, Confirmatory factor analysis, China

## Abstract

**Background:**

To the best of our knowledge, although the Chinese version of the Yale Global Tic Severity Scale (YGTSS) is widely used in child psychiatry departments in China, there is very little evidence focusing on the psychometric characteristics of the Chinese version of YGTSS. In this present study, we aim to re-examine the structure of the Chinese version of YGTSS and investigate its reliability and validity.

**Methods:**

A total of 367 children and adolescents with tic disorders aged 5–16 years old participated in the study. The Cronbach’s alpha, test-retest reliability and concurrent validity will be calculated. Confirmatory Factor Analysis (CFA) also will be performed to assess the structure of YGTSS.

**Results:**

The Cronbach’s alpha of the motor tic subscale of YGTSS was 0.84, for the phonic tic subscale of YGTSS, it was 0.90, but for the whole scale, it was 0.58. The test-retest reliability of YGTSS was 0.84. For the results of CFA, the Comparative Fit Index (CFI) of YGTSS based on the Two-Factor Model and Three-Factor Model was 0.97 and 0.96 respectively. The measurement invariance analysis suggested that the Two-Factor model of YGTSS across different age and sex groups was at the accepted level (≥0.90).

**Conclusion:**

Overall, according to the results of this research, it suggested that the Chinese version of YGTSS showed good psychometric properties. It can be used in the assessment of tic disorders in the Chinese population. In the future, more comprehensive tools for assessing tics need to be further developed, which can cover the symptoms of premonitory urge and tic related obsessive-compulsive symptoms.

**Supplementary Information:**

The online version contains supplementary material available at 10.1186/s12888-021-03399-5.

## Background

Tic disorders include Tourette Disorder, Persistent (Chronic) Motor or Phonic Tic Disorder and Provisional Tic Disorder [1]. The cardinal feature of tic disorders is rapid, recurrent, non-rhythmic movements or vocalizations [2, 3]. It should be noted that tic symptoms involve multiple dimensions (such as the frequency, severity) and a variety of accompanying symptoms (such as the premonitory urge and obsessive-compulsive symptoms) [4]. Therefore, an accurate and comprehensive assessment tool of tic symptoms is essential for the clinical management of tic disorders [5–8].

For the assessment of tic symptoms, there are several screening instruments for tics and associated symptoms have been developed [9–13]. Notably, the Yale Global Tic Severity Scale (YGTSS) is a commonly used tool for assessing the severity of tic symptoms among children and adolescents with tic disorders [12]. The YGTSS is a semi-structured clinician-assessed instrument that assesses the number, frequency, intensity, complexity and interference of the motor and phonic tics, as well as the impairment of tic symptoms [8, 14]. It was developed by Leckman in 1989 and it was widely used for the assessment of tic symptoms all over the world which also included China [15]. It was reported that there were several strengths of YGTSS: first, it can comprehensively evaluate the severity of the tic symptoms based on five dimensions mentioned above, which helps to characterize and quantify the tic symptoms; second, YGTSS is sensitive to the treatment response and is also widely used in clinical and scientific research in tic related disorder such as the tic-related obsessive-compulsive disorders; thirdly, it showed “stable” structure and good psychometric characteristics [16–19].

For the psychometric properties of YGTSS, several studies from different areas have proved that it showed excellent internal consistency and good test-retest reliability [15–17, 20]. The Two-Factor (motor and phonic) structure of YGTSS was also supported [15, 16, 21]. However, there few studies focus on the psychometric properties of the Chinese version of YGTSS. As far as we know, the first report on the use of YGTSS in China was in 2006 [22]. However, the sample size of this study is small (only 72 patients were included), and only an internal consistency coefficient was reported. Moreover, the sample included in this study was investigated before 14 years. It should be noted that, during the past 14 years, the criteria of diagnosis for tic disorders have undergone huge development [23]. It indicated that the psychometric properties of the Chinese version of YGTSS needed to be updated. Based on the statements above, it suggests that there is an urgent need to re-examine the structure of the Yale Global Tic Severity Scale in a large sample of children with tic disorders in China.

Therefore, this study aimed to measure the psychometric properties of the Chinese version of YGTSS. For the reliability of YGTSS, the Cronbach’s alpha, test-retest reliability will be calculated, while for the validity of YGTSS, the concurrent validity and Confirmatory Factor Analysis (CFA) will be performed.

## Method

### Participants

All patients included were outpatients in the Pediatric Psychiatric Department of Beijing Children’s Hospital from January 1, 2019, to May 1, 2020. We used the following inclusion criteria: (i) TD diagnosis that meets the Diagnostic and Statistical Manual of Mental Disorders, Fifth Edition (DSM-5) [24]. Including three types of tic disorder; (ii) The age range is 5–16 years old. Patients suffering from other mental disorders (i.e. autistic spectrum disorder, intellectual disability and depression), other movement disorders, epilepsy, acute febrile disease, malignancy, autoimmune diseases, history of head trauma with loss of consciousness, ingestion of drugs (except for patients with comorbidities, drugs that can significantly change the excitability of the cortex) were excluded from the study.

Finally, a total of 367 participants were included in this study. This study was approved by the Ethics Committee of Beijing Children’s Hospital Affiliated to Capital Medical University, and the written informed consent of the participant’s guardian was obtained.

Some studies have proved that age is an important factor in the pathogenesis and clinical manifestations of tic [25–28]. TS follows a time course of development, during which tics usually become more and more controlled during adolescence. It has been reported that the severity of tics is changing with the age and the age of 10 years in a critical cutoff [11]. To evaluate the effectiveness and reliability of YGTSS at different ages, the “Older group” (aged > 10 years, *n* = 83) and the “Younger group” (aged ≤10 years, *n* = 284) were defined.

### Measures

#### Yale global tic severity scale (YGTSS)

YGTSS is a scale assessed by clinicians, with proven reliability and validity, designed to measure the severity of tics in the previous week [15, 16]. The initial part of YGTSS includes 40 possible twitch checklists, which are divided into the simple motor, complex motor, simple phonic and complex phonic. Different types of simple phonic tics (such as coughing, throat clearing, sniffing, grunting, animal noises) were grouped into one category, called any simple Phonic tic. The tics that occurred in the past week were then overall scored on a series of 5-point scales (number, frequency, intensity, complexity, and inference), while motor and phonic tics were scored separately. YGTSS obtained three tic severity scores: Total Motor (0–25); Total Phonic (0–25) and the combined Total Tic Score (0–50). In addition, the YGTSS also includes an Impairment scale scored from 0 to 50 [15].

#### Premonitory urge for tics scale (PUTS)

Across participants, PUTS was used to evaluate premonitory urges [11]. PUTS is a self-report questionnaire that contains nine items to assess the presence and current degree of premonitory sensations in patients with chronic tic disorders. The total score ranges from 9 to 36 [29].

#### Children’s Yale-Brown obsessive-compulsive scale (CY-BOCS)

Using the Children’s Yale-Brown Obsessive-Compulsive Scale assessed the obsessive-compulsive symptom severity (CY-BOCS; Centers for Disease Control, 2009). CY-BOCS is a semi-structured interview tool managed by clinicians to measure the severity of the obsessive-compulsive disorder. It included 10 items with total severity scores ranging between 0 and 40. The CY-BOCS has showed demonstrated reliability and validity [30].

Besides, a total of four raters (FW, YG, JL, and LY) were trained to complete the measures assessed by clinicians. The inter-correlation coefficient (ICC) of these raters was 0.87. In order to check the test-retest reliability of the YGTSS, a total of 30 participants were selected randomly to finish the re-test of YGTSS after 1 month.

#### Data analysis

All analyses were performed using the Windows social science statistical software package (SPSS Inc., Chicago, IL, USA, v25.0). Pearson’s correlation was used to examine the item-total correlation of YGTSS. Cronbach’s alpha is a measure of internal consistency. The test-retest reliability was calculated to test the reliability of YGTSS. Also, the CFA was performed to test the structure of YGTSS in R (version 3.5.3) using the package “Lavaan”.

In addition, measurement invariance is often tested in the framework of multiple-group, there are four types of measurement invariance: (1) configural, equivalence of model form; (2) metric (weak factorial), equivalence of factor loadings; (3) scalar (strong factorial), equivalence of item intercepts or thresholds; and (4) residual (strict or invariant uniqueness), equivalence of items’ residuals or unique variances. The fit indexes are Root Mean Square Error of Approximation (RMSEA), Standardized Root Mean-square Residual (SRMR), Comparative Fit Index (CFI), Tucker-Lewis Index (TLI), etc. (acceptable criteria: RMSEA< 0.08, SRMR< 0.08, CFI ≥ 0.90, TLI > 0.90) [31].

## Results

### Descriptive data

A total of 367 children and adolescents with tic disorders were included in this study. The average age of them is 9.22 ± 2.06 years, the age of onset is 7.28 ± 2.13 years, and the average duration of illness is 1.93 ± 1.72 years.

The total score range of YGTSS in children with tic was 6–63, with an average score of 21.64 ± 8.95, among which, the score range of motor tic was 0–22, with an average score of 12.78 ± 4.07, the score range of phonic tic was 0–19, with an average score of 6.25 ± 5.55, the score range of functional impairment was 0–30, with an average score of 2.64 ± 4.88. Moreover, the score range of PUTS was 0–30, with an average PUTS score of 12.81 ± 3.17. The score range of CY-BOCS total was 0–17, the average score was 3.93 ± 5.15. For more details, see Table 1. In addition, the correlation analysis between the YGTSS and PUTS showed the correlation coefficient between them was 0.12 (*p* < 0.05), while the YGTSS and CY-BOCS were 0.23 (*p* < 0.01). For more details, see Table 2.

**Table 1:** The Clinical Characteristics for Total Sample and Different Group (*N* = 367)

	Min	Max	Mean	SD	Skewness	Kurtosis
**Age**	5	16	9.22	2.056	0.586	−0.004
**Age of illness**	2	14	7.28	2.130	0.383	0.063
**DOI**	0.04	9	1.93	1.72	1.158	0.945
**Motor Numbers**	0	5	3.81	1.135	−1.909	4.439
**Motor Frequency**	0	5	3.51	1.377	−1.079	0.386
**Motor Intensity**	0	5	2.66	0.844	−1.474	3.189
**Motor Complexity**	0	5	1.80	1.194	0.042	−1.309
**Motor Interference**	0	4	1.01	0.401	1.340	12.159
**Motor Total**	0	22	12.78	4.069	−1.505	3.028
**Phonic Numbers**	0	5	1.94	1.700	−0.113	−1.688
**Phonic Frequency**	0	5	1.80	1.793	0.351	−1.475
**Phonic Intensity**	0	4	1.58	1.396	−0.052	−1.647
**Phonic Complexity**	0	4	0.34	0.586	2.301	8.438
**Phonic Interference**	0	3	0.60	0.544	0.224	−0.305
**Phonic Total**	0	19	6.25	5.546	−0.024	−1.579
**Motor and photic Total**	6	38	19.03	6.501	0.224	−0.904
**Impairment Total**	0	30	2.64	4.886	1.766	3.183
**YGTSS Total**	6	63	21.64	8.954	0.885	1.362
**PUTS Total**	9	26	12.81	3.169	0.533	0.425
**Compulsive Behaviors**	0	17	2.68	3.726	0.961	−0.373
**Obsessive Thoughts**	0	14	1.30	2.906	2.239	4.120
**CY-BOCS Total**	0	27	3.93	5.153	1.335	1.787

Note: *Min* Minimum, *Max* Maximum, *SD* Standard Deviation, *DOI* Duration of Illness, *YGTSS Motor Score* Yale Global Tic Severity Scale Total Motor Tic Score, *YGTSS Phonic Score* Yale Global Tic Severity Scale Total Phonic Tic Score, *YGTSS Total Score* Yale Global Tic Severity Scale Global Severity Score, *Impairment Score* Yale Global Tic Severity Scale Overall Impairment Score, *PUTS Total* Premonitory Urge for Tics Scale Total Score, *CY-BOCS Total* Children’s Yale-Brown Obsessive Compulsive Scale Total Score

**Table 2:** Correlation Analysis of YGTSS Scores with PUTs and CY-BOCS

	Motor Total	Phonic Total	Motor & photic Total	Impairment total	YGTSS Total	PUTS Total	Compulsive Behaviors	Obsessive Thoughts	CY-BOCS Total
**Motor Total**	**–**	−.112*	.530**	.161**	.471**	**.032**	**−.019**	**.095**	**.051**
**Phonic Total**	**–**	**–**	.783**	.139**	.648**	**.056**	.103*	.124*	.135**
**Motor and photic Total**	**–**	**–**	**–**	.220**	.848**	**.068**	**.077**	.165**	.147**
**Impairment total**	**–**	**–**	**–**	**–**	.701**	.126*	.159**	.175**	.219**
**YGTSS Total**	**–**	**–**	**–**	**–**	**–**	.122*	.145**	.212**	.225**
**PUTS Total**	**–**	**–**	**–**	**–**	**–**	**–**	**.035**	.154**	.112*
**Compulsive Behaviors**	**–**	**–**	**–**	**–**	**–**	**–**	**–**	.191**	.822**
**Obsessive Thoughts**	**–**	**–**	**–**	**–**	**–**	**–**	**–**	**–**	.707**

*Note: Min* minimum, *Max* Maximum, *SD* Standard Deviation, *DOI* Duration of Illness, *YGTSS Motor Score* Yale Global Tic Severity Scale Total Motor Tic Score, *YGTSS Phonic Score* Yale Global Tic Severity Scale Total Phonic Tic Score, *YGTSS Total Score* Yale Global Tic Severity Scale Global Severity Score, *Impairment Score* Yale Global Tic Severity Scale Overall Impairment Score, *PUTS Total* Premonitory Urge for Tics Scale Total Score, *CY-BOCS Total* Children’s Yale-Brown Obsessive Compulsive Scale Total Score; *: p < 0.05. **: p < 0.01. Pearson r correlations are at the p < 0.05 level

### Validity of the YGTSS

**Fig. 1:**
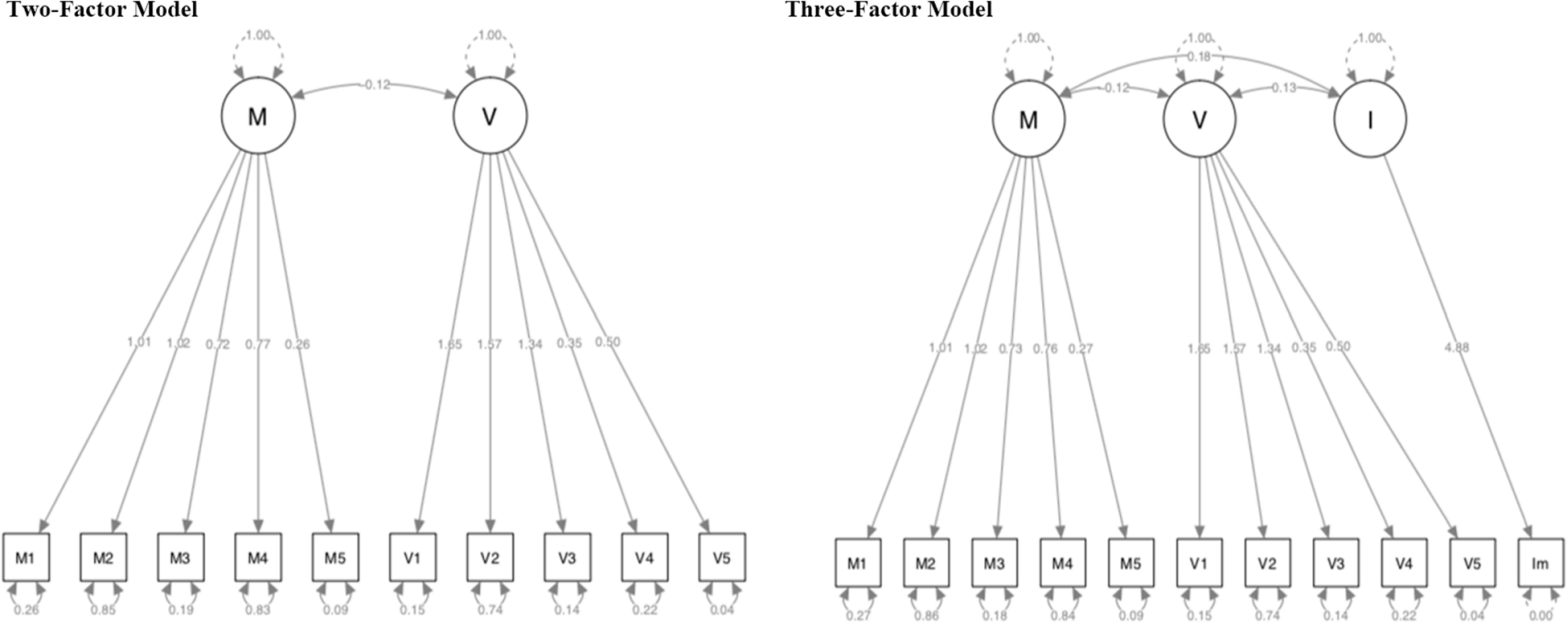
The Two-Factor Model and Three-Factor Model for the CFA of YGTSS (M: Motor subscale; P: Phonic subscale; I: Impairment subscale)

The results of CFA for YGTSS showed that the Comparative Fit Index (CFI) and Tucker-Lewis Index (TLI) based on the Two-Factor Model was 0.97 and 0.96 separately, while the Three-Factor Model (motor factor, phonic factor and impairment) was 0.96 and 0.95 separately. Akaike (AIC) and Sample-size adjusted Bayesian (BIC) based on Two-Factor Model were 7326.45 and 7341.84 separately, while the Three-Factor Model was 9518.98 and 9536.57 separately. Root Mean Square Error of Approximation (RMSEA) and Standardized Root Mean Square Residual (SRMR) was 0.08 and 0.03 separately, while the Three-Factor Model was 0.09 and 0.04 separately. For more details, see Fig. 1& Table 3.

**Table 3:** The results of CFA for Different Groups (*N* = 367)

Model	Variable	Younger Group	Older Group	Male Group	Female Group	Total
Two-factor model	CFI	0.969	0.967	0.972	0.952	0.972
	TLI	0.959	0.956	0.963	0.937	0.963
	AIC	5537.337	1759.21	5881.455	1418.627	7326.45
	BIC	5547.373	1743.766	5892.143	1400.834	7341.837
	RMSEA	0.088	0.089	0.081	0.12	0.083
	SRMR	0.036	0.052	0.038	0.066	0.034
Three-factor model	CFI	0.962	0.949	0.963	0.935	0.962
	TLI	0.951	0.934	0.951	0.915	0.95
	AIC	7143.35	2300.536	7651.142	1842.883	9518.982
	BIC	7154.82	2282.886	7739.466	1822.548	9536.568
	RMSEA	0.088	0.1	0.085	0.127	0.088
	SRMR	0.041	0.059	0.043	0.071	0.04

*Note: CFI* Comparative Fit Index, *TLI* Tucker-Lewis Index, *AIC* Akaike, *BIC* Sample-size adjusted Bayesian, *RMSEA* Root Mean Square Error of Approximation, *SRMR* Standardized Root Mean Square Residual, *ICC* Inter-correlation Coefficient

### The results of CFA for different groups

We have done measurement invariance of the models, to see CFA and fit data for each group for age younger vs. older and male vs. female. The results of CFA for YGTSS showed that regardless of the sex group and different age groups, their two-factor and three-factor models are stable. See Table 3 for specific parameters.

In order to test the structure of YGTSS across different groups, we also calculated the measurement invariance including the “configural invariance”, “metric invariance”, “scalar invariance”, and “strict invariance”. The two-factor model of YGTSS was tested by measurement invariance based on the “age” groups and “sex” groups. The four types of measurement invariance for two-factor model were all at the accepted level. For more details, see Table 4.

**Table 4:** Measurement Invariance for Different Groups (*N* = 367)

	Model	χ2 (df)	CFI	TLI	AIC	BIC	RMSEA (90% CI)	SRMR	Decision
**Basic model**	**–**	**–**	**0.972**	**0.963**	**7326.450**	**7341.837**	**0.083**	**0.034**	**Accept**
**Age Model**	**Basic model**	**–**	**0.969**	**0.958**	**7336.546**	**7578.679**	**0.088**	**0.037**	**Accept**
	**Configural Invariance**	**68**	**1.000**	**0.999**	**–**	**–**	**0.009(0.000–0.045)**	**0.036**	**Accept**
	**Scalar Invariance**	**76**	**0.996**	**0.996**	**–**	**–**	**0.022(0.000–0.049)**	**0.045**	**Accept**
	**Metric Invariance**	**84**	**0.996**	**0.996**	**–**	**–**	**0.021(0.000–0.047)**	**0.046**	**Accept**
	**Strict invariance**	**94**	**0.995**	**0.995**	**–**	**–**	**0.023(0.000–0.047)**	**0.056**	**Accept**
**Gender Model**	**Basic model**	**–**	**0.967**	**0.956**	**7340.083**	**7582.215**	**0.091**	**0.040**	**Accept**
	**Configural Invariance**	**68**	**0.996**	**0.994**	**–**	**–**	**0.024(0.000–0.051)**	**0.039**	**Accept**
	**Scalar Invariance**	**76**	**0.995**	**0.994**	**–**	**–**	**0.025(0.000–0.050)**	**0.043**	**Accept**
	**Metric Invariance**	**84**	**0.996**	**0.996**	**–**	**–**	**0.021(0.000–0.047)**	**0.044**	**Accept**
	**Strict invariance**	**94**	**0.995**	**0.995**	**–**	**–**	**0.023(0.000–0.047)**	**0.053**	**Accept**

*Note: age model: N = 367, Younger group n = 284, Older group n = 83; gender model: N = 367, Male group n = 293, Female group n = 74; CFI* Comparative Fit Index, *TLI* Tucker-Lewis Index, *AIC* Akaike, *BIC* Sample-size adjusted Bayesian, *RMSEA* Root Mean Square Error of Approximation, *SRMR* Standardized Root Mean Square Residual

### Age and sex differences of YGTSS, PUTS and CY-BOCS

The average YGTSS total score of the Younger group was 20.64 ± 7.90, and the Older group was 25.07 ± 11.28, the difference between the two groups was statistically significant (t = − 2.54, *p* < 0.05). The mean score of the Younger group and Older group of Impairment total was 2.01 ± 4.10 and 4.82 ± 6.51 respectively, with statistical significance (t = − 3.73, *p* < 0.01). The mean CY-BOCS total score of the Younger group and Older group were 3.31 ± 4.53 and 6.02 ± 6.47 respectively, the difference between the two groups was statistically significant(t = − 3.57, *p* < 0.01).

The YGTSS total average score of the male group was 22.46 ± 8.96, the female group was 18.41 ± 8.23, the difference between the two groups was statistically significant (t = 3.54, *p* < 0.01). The Impairment total score of the male group and female group was 2.87 ± 5.03 and 1.76 ± 4.17, respectively, and there was no significant difference between them. The PUTS average score of the male group was 13.00 ± 3.20, and the female group was 12.07 ± 2.94. the difference between the two groups was statistically significant (t = 2.27, *p* < 0.05). For more details, see Table 5.

**Table 5:** The Clinical Characteristics for Different Age & Gender Groups (*N* = 367)

Groups	Younger Group Mean (SD)	Older Group Mean (SD)	***P Value*** (Younger vs. Older)	Male Group Mean (SD)	Female Group Mean (SD)	***P Value*** (Male vs. Female))
**Motor Total**	**12.49(4.090)**	**13.77(3.855)**	−2.536*	**13.08(3.840)**	**11.61(4.719)**	2.804**
**Phonic Total**	**6.18(5.486)**	**6.48(5.775)**	**−0.442**	**6.52(5.522)**	**5.18(5.548)**	**1.863**
**Motor and phonic Total**	**18.67(6.264)**	**20.25(7.214)**	**−1.96**	**19.59(6.425)**	**16.78(6.355)**	3.369**
**Impairment total**	**2.01(4.099)**	**4.82(6.507)**	−3.727**	**2.87(5.031)**	**1.76(4.174)**	**1.751**
**YGTSS Total**	**20.64(7.897)**	**25.07(11.282)**	− 3.347**	**22.46(8.957)**	**18.41(8.231)**	3.535**
**PUTS Total**	**12.63(3.024)**	**13.41(3.579)**	**−1.969**	**13.00(3.202)**	**12.07(2.944)**	2.266*
**Compulsive Behaviors**	**2.34(3.505)**	**3.84(4.218)**	−2.966**	**2.74(3.819)**	**2.43(3.348)**	**0.635**
**Obsessive Thoughts**	**1.04(2.534)**	**2.18(3.810)**	−2.570**	**1.31(2.906)**	**1.26(2.924)**	**0.133**
**CY-BOCS Total**	**3.31(4.533)**	**6.02(6.468)**	−3.571**	**3.99(5.215)**	**3.69(4.927)**	**0.443**

*Note: SD* Standard Deviation, *YGTSS Motor Score* Yale Global Tic Severity Scale Total Motor Tic Score, *YGTSS Phonic Score* Yale Global Tic Severity Scale Total Phonic Tic Score, *YGTSS Total Score* Yale Global Tic Severity Scale Global Severity Score, *Impairment Score* Yale Global Tic Severity Scale Overall Impairment Score, *PUTS Total* Premonitory Urge for Tics Scale Total Score, *CY-BOCS Total* Children’s Yale-Brown Obsessive Compulsive Scale Total Score, *N/A* Not applicable; *: p < 0.05. **: p < 0.01

### Reliability of the YGTSS

For the Cronbach’s alpha of YGTSS, the Total Motor Tic Score was 0.84, Total Phonic Tic Score was 0.90, but for the Total Tic Score, it was 0.58. The test-retest reliability (1 month) was 0.84. In order to investigate the internal consistency of the scores of each item, the correlation among items, subscales and overall scores was calculated. The Pearson correlations between the YGTSS item scores and the corresponding subscale score, as well as with the overall impairment and global tic scores were presented. It turns out that each item score is highly correlated with its corresponding subscale score (*p* < 0.05). For more details, see sTable 1 in the Supplementary Material.

## Discussion

The preliminary data provided in this report indicate that YGTSS is an effective tool for tic symptoms assessment [12]. Although YGTSS is widely used in China to evaluate the clinical tic symptoms and treatment effects of tics and it was regarded as the primary outcome index [32–34], there is no evidence for its psychometric properties. This study provides psychometric evidence of the Chinese version of YGTSS in children and adolescents with tic disorders and shows good reliability and validity.

In the present study, it found goodness-of-fit indices were high for both the two-factor model and the three-factor model of YGTSS. In order to further analyze the goodness of fit index of YGTSS’s two-factor model and three-factor model, we made a comparison between different age groups and sex groups and found that the YGTSS two-factor model and three-factor model in different age and sex groups Goodness of fit index is very high. But it has been reported that the goodness-of-fit indices were not high for the three-factor model [15, 20]. The possible reasons need to be further explored in future. In this present study, we found that the internal consistency reliability scores of the Motor scale and Phonic scale are high, but after adding the Impairment subscale, the internal consistency reliability score of YGTSS Total was decreased. Although the Impairment subscale is also an important dimension of YGTSS [15], two issues need to be addressed in future. First, the content of functional impairment is not clear, which might confuse raters to finish the assessment. Second, the item’s score weight is relatively large, which is equivalent to the sum of the highest motor and phonic factor scores. Therefore, it suggests that in the development of a revised version of YGTSS in the future, functional impairment needs to be evaluated in more detailed items, and appropriate score weight is assigned.

This study analyzed the relationship between YGTSS and CY-BOCS and PUTS. We found that the severity of tics has a slight to moderate positive correlation with premonitory urge (PU) and obsessive-compulsive symptoms (OCS). Notably, it found that the correlation coefficient between the severity of tics and the severity of OCS is significant [35]. It is worth noting that 90% of TS patients suffer from other mental illnesses, the most common is obsessive-compulsive disorder [36–38]. Previous studies have also confirmed that the severity of the premonitory urge can predict the severity of OCS in TS [4]. Furthermore, in our previous meta-analysis, we also found a correlation between the severity of PU and tic symptoms [39]. In general, PU and OCS can reflect the severity of tic symptoms. These two type of symptoms might also the core symptoms of tic disorders. Based on these results in this present study, it further confirms the connection between the three types of symptoms (Tic, PU and OCS).

In addition, among patients diagnosed with childhood obsessive-compulsive disorder, 10–40% of patients may develop the subtype of obsessive-compulsive disorder related to tics in the fifth edition of the Diagnostic and Statistical Manual of Mental Disorders (DSM-5) [1, 40], and approximately 25–50% of patients with TS meet criteria for OCD [14, 41]. For these subtype of tic related disorders, the YGTSS might not cover all mentions of these symptoms. It indicated that we can develop some more comprehensive assessment tools for the assessment of tic and tic-related disorders in the future, which can cover the related symptoms such as the PU and OCS.

Overall, the YGTSS is a useful and basic instrument to measure changes in tic symptoms in different course and setting. It can be widely used in the assessment of children and adolescents with tic disorders in China. For the future development of YGTSS, two issues need to be addressed. First, the subscale of the functional impairment of YGTSS need further exploration or revision; Second, tic related symptoms (such as PU and OCS) are also very important dimensions of TS which can be included in a future version of YGTSS.

Three limitations of this study need to be considered. First, these participants were recruited by children and adolescents but without adults which may not generalize to the wider populations with TD. Second, because there are no other Chinese version scales for the severity of tic symptoms in China, no tools were used for the calculation of concurrent validity of YGTSS. Third, the sample size is still small, a large sample size was needed to confirm these results.

## Conclusion

In conclusion, YGTSS has good psychometric properties in Chinese children and adolescents with tic disorders. No matter the two-factor model and the three-factor model was found the goodness-of-fit. YGTSS can be widely used in the evaluation of Chinese tic patients. In the future, more comprehensive tools for the assessments of tic symptoms need to be developed.

## Supplementary Information

**Additional file 1: Table S1**. Correlations between YGTSS Item Scores (*N* = 367).

## Data Availability

The datasets used and/or analyzed during the current study are available from the corresponding author on reasonable request.

## Abbreviations

YGTSS: Yale Global Tic Severity Scale
TD: Tic Disorders
DSM-5: The fifth edition of the Diagnostic and Statistical Manual of Mental Disorders
OCS: Obsessive-compulsive Symptoms
OCD: Obsessive-compulsive Disorders
CFI: Comparative Fit Index
TLI: Tucker-Lewis Index
AIC: Akaike
BIC: Sample-size adjusted Bayesian
RMSEA: Root Mean Square Error of Approximation
SRMR: Standardized Root Mean Square Residual
ICC: Inter-correlation Coefficient
PUTS: Premonitory Urge for Tics Scale
PU: Premonitory Urge
CY-BOCS: Children’s Yale-Brown Obsessive-Compulsive Scale
CFA: Confirmatory Factor Analysis

## References

[1] APA APA (2013). Diagnostic and statistical manual of mental disorders.

[2] Tamara P (2013). Tourette syndrome and other tic disorders of childhood. Handb Clin Neurol.

[3] Jankovic J, Kurlan R (2011). Tourette syndrome: evolving concepts. Mov Disord.

[4] Yan J, Yu L, Wen F, Wang F, Liu J, Cui Y (2020). The severity of obsessive-compulsive symptoms in Tourette syndrome and its relationship with premonitory urges: a meta-analysis. Expert Rev Neurother.

[5] Ganos C, Martino D (2015). Tics and tourette syndrome. Neurol Clin.

[6] Cavanna AE, Rickards H (2013). The psychopathological spectrum of Gilles de la Tourette syndrome. Neurosci Biobehav Rev.

[7] Hirschtritt ME, Lee PC, Pauls DL, Dion Y, Grados MA, Illmann C (2015). Lifetime prevalence, age of risk, and genetic relationships of comorbid psychiatric disorders in Tourette syndrome. JAMA Psychiatry.

[8] McGuire JF, Piacentini J, Storch EA, Murphy TK, Ricketts EJ, Woods DW (2018). A multicenter examination and strategic revisions of the Yale global tic severity scale. Neurology.

[9] Schmidt C, Saadioui M, Bohmer V, Host V, Spirlet MR, Desreux JF (2003). Modification of calix[4] arenes with CMPO-functions at the wide rim. Synthesis, solution behavior, and separation of actinides from lanthanides. Org Biomol Chem.

[10] Storch EA, Murphy TK, Geffken GR, Soto O, Sajid M, Allen P (2004). Further psychometric properties of the Tourette's disorder scale-parent rated version (TODS-PR). Child Psychiatry Hum Dev.

[11] Woods DW, Piacentini J, Himle MB, Chang S (2005). Premonitory urge for tics scale (PUTS): initial psychometric results and examination of the premonitory urge phenomenon in youths with tic disorders. J Dev Behav Pediatr.

[12] Martino D, Pringsheim TM, Cavanna AE, Colosimo C, Hartmann A, Leckman JF (2017). Systematic review of severity scales and screening instruments for tics: critique and recommendations. Mov Disord.

[13] Robertson MM, Banerjee S, Kurlan R, Cohen DJ, Leckman JF, McMahon W (1999). The Tourette syndrome diagnostic confidence index: development and clinical associations. Neurology.

[14] Cohen SC, Leckman JF, Bloch MH (2013). Clinical assessment of Tourette syndrome and tic disorders. Neurosci Biobehav R.

[15] Leckman JF, Riddle MA, Hardin MT, Ort SI, Swartz KL, Stevenson J (1989). The Yale global tic severity scale: initial testing of a clinician-rated scale of tic severity. J Am Acad Child Adolesc Psychiatry.

[16] Storch EA, Murphy TK, Geffken GR, Sajid M, Allen P, Roberti JW (2005). Reliability and validity of the Yale global tic severity scale. Psychol Assess.

[17] Stefanoff P, Wolanczyk T (2005). Validity and reliability of polish adaptation of Yale global tic severity scale (YGTSS) in a study of Warsaw schoolchildren aged 12-15. Przegl Epidemiol.

[18] Kircanski K, Woods DW, Chang SW, Ricketts EJ, Piacentini JC (2010). Cluster analysis of the Yale global tic severity scale (YGTSS): symptom dimensions and clinical correlates in an outpatient youth sample. J Abnorm Child Psychol.

[19] Kloft L, Steinel T, Kathmann N (2018). Systematic review of co-occurring OCD and TD: evidence for a tic-related OCD subtype?. Neurosci Biobehav Rev.

[20] Storch EA, Murphy TK, Fernandez M, Krishnan M, Gefficen GR, Kellgren AR (2007). Factor-analytic study of the Yale global tic severity scale. Psychiatry Res.

[21] Garcia-Lopez R, Perea-Milla E, Romero-Gonzalez J, Rivas-Ruiz F, Ruiz-Garcia C, Oviedo-Joekes E (2008). Spanish adaptation and diagnostic validity of the Yale global tics severity scale. Rev Neurol.

[22] Zhong YQ, Wu J, Xie XL, Hu WG, Zhou WZ (2006). The introduction of Yale Globle tic severity scale into the clinical evaluation of children with tic disorders. Chin J Prac Pediatr.

[23] Hirschtritt ME, Dy ME, Yang KG, Scharf JM (2016). Child neurology: diagnosis and treatment of Tourette syndrome. Neurology.

[24] Roessner V, Hoekstra PJ, Rothenberger A (2011). Tourette's disorder and other tic disorders in DSM-5: a comment. Eur Child Adolesc Psychiatry.

[25] Groth C, Mol Debes N, Rask CU, Lange T, Skov L (2017). Course of Tourette syndrome and comorbidities in a large prospective clinical study. J Am Acad Child Adolesc Psychiatry.

[26] Peterson BS, Pine DS, Cohen P, Brook JS (2001). Prospective, longitudinal study of tic, obsessive-compulsive, and attention-deficit/hyperactivity disorders in an epidemiological sample. J Am Acad Child Adolesc Psychiatry.

[27] Leckman JF, Peterson BS, Pauls DL, Cohen DJ (1997). Tic disorders. Psychiatr Clin North Am.

[28] Pepes SE, Draper A, Jackson GM, Jackson SR (2016). Effects of age on motor excitability measures from children and adolescents with Tourette syndrome. Dev Cogn Neurosci.

[29] Brandt VC, Beck C, Sajin V, Anders S, Munchau A (2016). Convergent validity of the PUTS. Front Psychiatry.

[30] Storch EA, Murphy TK, Bagner DM, Johns NB, Baumeister AL, Goodman WK (2006). Reliability and validity of the child behavior checklist obsessive-compulsive scale. J Anxiety Disord.

[31] Putnick DL, Bornstein MH (2016). Measurement invariance conventions and reporting: the state of the art and future directions for psychological research. Dev Rev.

[32] Gu Y, Li Y, Cui Y (2020). Correlation between premonitory urges and tic symptoms in a Chinese population with tic disorders. Pediatr Investig.

[33] Liu ZS, Cui YH, Sun D, Lu Q, Jiang YW, Jiang L (2020). Current status, diagnosis, and treatment recommendation for tic disorders in China. Front Psychiatry.

[34] Zhao X, Wang S, Hao J, Zhu P, Zhang X, Wu M (2020). A whole-exome sequencing study of Tourette disorder in a Chinese population. DNA Cell Biol.

[35] Kano Y, Kono T, Matsuda N, Nonaka M, Kuwabara H, Shimada T (2015). The impact of tics, obsessive-compulsive symptoms, and impulsivity on global functioning in Tourette syndrome. Psychiatry Res.

[36] Lombroso PJ, Scahill L (2008). Tourette syndrome and obsessive-compulsive disorder. Brain and Development.

[37] Mula M, Cavanna AE, Critchley H, Robertson MM, Monaco F (2008). Phenomenology of obsessive compulsive disorder in patients with temporal lobe epilepsy or tourette syndrome. J Neuropsychiatr Clin Neurosci.

[38] Eddy CM, Cavanna AE (2014). Premonitory urges in adults with complicated and uncomplicated Tourette syndrome. Behav Modif.

[39] Li Y, Wang F, Liu J, Wen F, Yan C, Zhang J (2019). The correlation between the severity of premonitory urges and tic symptoms: a meta-analysis. J Child Adolesc Psychopharmacol.

[40] Leckman JE, Denys D, Simpson HB, Mataix-Cols D, Hollander E, Saxena S (2010). Obsessive-compulsive disorder: a review of the diagnostic criteria and possible subtypes and dimensional specifiers for Dsm-V. Depress Anxiety.

[41] Lebowitz ER, Motlagh MG, Katsovich L, King RA, Lombroso PJ, Grantz H (2012). Tourette syndrome in youth with and without obsessive compulsive disorder and attention deficit hyperactivity disorder. Eur Child Adolesc Psychiatry.

