# Supplementary material for: Revisiting the structure of the Yale Global Tic Severity Scale (YGTSS) in a sample of Chinese children with tic disorders
Source: BMC Psychiatry. 2021 Aug 9;21:394. doi: 10.1186/s12888-021-03399-5 (PMC8351146; doi:10.1186/s12888-021-03399-5)
Supplement: Supplementary file 1 — Additional file 1: Table S1. Correlations between YGTSS Item Scores (N = 367). [file 12888_2021_3399_MOESM1_ESM.docx]

**sTable 1** **Correlations between YGTSS Item Scores (N = 367)**

|  | **Motor Total** |  | **Phonic Total** |  | **Impairment Total** |
| --- | --- | --- | --- | --- | --- |
| **Motor** | **N/A** | **Phonic** | **N/A** | **Motor Total** | **0.156**** |
| **Numbers** | **0.783*** | **Frequency** | **0.942**** | **Phonic Total** | **0.131*** |
| **Frequency** | **0.733*** | **Frequency** | **0.959**** | **YGTSS Total** | **0.701**** |
| **Intensity** | **0.698*** | **Intensity** | **0.916**** | **N/A** | **N/A** |
| **Complexity** | **0.844*** | **Complexity** | **0.724**** | **N/A** | **N/A** |
| **Interference** | **0.424**** | **Interference** | **0.881**** | **N/A** | **N/A** |

*Note: Pearson r correlations are at the p < 0.05 level. *: p <0.05, **: p <0.01. N/A: Not Applicable.*
